# 

**Published:** 1916-07-15

**Authors:** 


### Australian Hospitals and the State.

An article in the *Medical Journal of Australia* suggests that more complete control of hospitals in Australia is sought by the State. The Labour Party, who are largely in power, claim that the masses have a right to free medical and surgical attention under the most favourable conditions, and ignorantly make the assumption that maximum efficiency and economy can be attained when the State takes over the responsibility and control of all persons concerned in the care of the sick. As we in this country are fully aware, this idea is no new one; its exponents have, however, much more chance of pushing their arguments home in a land where the contributions of the Government form by far the greater part of the revenue of the principal hospitals. Large though this support is, our contemporary claims that in many cases it is insufficient, and that by means of financial starvation a political party seeks to push its propaganda. The *Medical Journal* naturally and properly regards this important subject from the point of view of the medical profession, and in supplying an instance of the more expensive nature of an institution wholly supported and managed by the State inquires "whether there is sufficient evidence in favour of the proposed new order of things to justify the public in assuming that efficiency would not be sacrificed by requiring hospital physicians and surgeons to supply to the rich and poor alike a commodity which yields to them their livelihood, but which they are prepared to give to those who cannot pay for it."

### A Municipal Mental Deficiency Home.

A distinct step of progress is reported from Leicester, where the Mayor, Alderman Jonathan North, J.P., recently accepted, "for the benefit of the citizens of Leicester," from a local committee, a completely equipped home for mental cases. This, it was stated, is the first instance of the establishment of a mental deficients' home under the direct control of a municipality. The presentation was made by Colonel Astley V. Clarke, the treasurer of "Sunnyholme," a home formerly carried on under voluntary auspices for mentally deficient girls. The home being now closed on the operation of the Mental Deficiency Act, the committee from funds in hand acquired a large house and garden of about one and a half acres, equipped it, and now formally handed it over to the Mayor for the service of the community, the general idea being to continue the "home" atmosphere as distinct from "institutional" treatment. Occupational training for the inmates will be provided in housework, laundry-work, rug-making, cane-work, etc., while the garden affords opportunities for horticultural training. Councillor Reynolds (chairman) paid tribute to the "Sunnyholme" Committee for their public-spirited action in spending their capital in the acquisition and equipment of the new home, which is to be handed over, free of debt and charge, to the Corporation.

### HOSPITAL RESULTS IN 1915.

**Royal Hospital for Sick Children, Glasgow.**—Ordinary income has increased by £1,794, and ordinary expenditure by £6,339. In consequence of a neighbouring Poor-Law institution being taken over for the use of wounded soldiers 145 parochial children were treated in the hospital, which is now carrying on its work in a new building.

**South London Hospital for Women.**—While the new hospital on Clapham Common has been building, the out-patient department in Newington Causeway has been responsible for most of the work or this our youngest hospital, and new patients have increased from 1,619 in 1913 to 5,765 in 1915. Most of the cases came from the surrounding districts. To the temporary in-patient department seventy-one patients were admitted. The total income was £1,747, and the total expenditure £1,553. Towards the new building the balance-sheet shows that there was in hand at the end of the year a sum of £8,944.

**The London Lock Hospital and Rescue Home.**—The importance of the work of this hospital has been emphasised by the Report of the Royal Commission on Venereal Diseases. The institution comprises the female hospital, the rescue home, and the male hospital. The average stay at the female hospital has fallen from 128 to 65 days, but the patients admitted in 1915 were 623, as against 343 in 1914. The male hospital statistics show little change. Total income (including £2,470 legacies and £6,535 patients' payments), £17,785. Total expenditure, £13,818.

**Adelaide Hospital, South Australia.**—The hospital being a Government department, all moneys received are paid into the Treasury, from which all disbursements are made; the local management is, however, much the same in form as in this country, and the medical and surgical services are provided by honorary visiting officers assisted by paid residents. The Inspector-General of Hospitals is the connecting link between the board of management and the Government. The expenditure for 1915 was £33,416, and to meet this the State provided £30,322, patients £1,462, and voluntary contributors £799, the balance being made up by students' fees, sales of drugs, etc. The accounts are not kept on the Uniform System.

**Royal Prince Albert Hospital, Sydney.**—The annual report is printed in the *Royal Prince Albert Hospital Gazette.* Of 6,683 patients admitted during 1915, 4,025 were accident or urgency and non-paying cases, and 2,658 contributed something towards their maintenance while in the hospital. Of an active honorary medical staff of forty, twelve are absent on war service. Although 150 beds had been offered for sick or wounded returned soldiers, no cases had been admitted up to the end of last year. The receipts amounted to £46,724, and of this sum £32,450 was provided by Government, patients contributed £6,289, and voluntary contributions amounted to £6,562. Of the total expenditure (£47,054) £22,691 was in respect of salaries and wages. The State is not the treasurer, as in the case of the Adelaide Hospital.

### WHAT'S WHAT COLUMN.

[*To meet the wants and wishes of many readers we have organised this Column. Its usefulness must depend upon its continuous use by Matrons, Sisters, and hospital workers. If full opportunity is taken of this Column the result may prove of world-wide importance.*]

### ANSWERS TO CORRESPONDENTS.

### Reading Case Reports.

*K. T.*—At this hospital the resident medical officer has instructed the sisters and nurses that they must not read the notes of the cases. Can you tell me if this is in accordance with ordinary hospital routine and if you consider it a wise measure?

We have never heard of any hospital making a rule that sisters and nurses should not read case notes, nor have the authorities of one of our greatest clinical hospitals either. We should imagine that such a rule would be likely to encourage a taste for literature in which in a general way sisters and nurses have little interest. Do the rules for the resident medical officer at the hospital referred to confer on him power to give any such order? If not, the matter is clearly one which should be brought before the medical board for report to the committee of management.

### Passing Events and Topics.

### Our Bank's Dividend.

THE directors of the London County and Westminster Bank, Limited, have declared an interim dividend of 9 per cent. for the half-year ending June 30. The dividend of 9s. per share (less income tax) will be payable on August 1.

### New President of West Ham Hospital.

THE Duchess of Marlborough, who for some time has been associated with the work of the West Ham and Eastern General Hospital, has now accepted the presidency in succession to Mr. C. E. Leo Lyle, who will remain chairman of the committee, having since Mr. T. A. Cook's vacation of that office held both offices.

### The Penalisation of Parenthood.

THE Eugenics Education Society has been devoting considerable attention to the question of reducing the present penalisation of parenthood. At the General Council Meeting, held on July 6, the following resolutions were unanimously adopted, and will be brought to the notice of those in authority:—(1) That it is desirable, without affecting the total amount raised by income-tax from the nation, to make changes in the incidence of the tax in order to decrease much further than has yet been done the present heavy burden on parents with dependent children. (2) That the best way of securing this adjustment would be to assess the total income of a family as a number of separate incomes, the number bearing some relation to the number of persons the income has to support. (3) That the executive committee be empowered to take such steps as may seem advisable to bring this resolution to the notice of the Government.

### Gifts to Hospitals.

LIVERPOOL CHILDREN'S INFIRMARY and the Royal Southern Hospital, Liverpool, each receives £900 under the will of Mr. Edward Wallace Black, of Liverpool, who died on March 9.

KING EDWARD VII.'s HOSPITAL, CARDIFF, has received towards the extension scheme 500 guineas from Messrs. James Howell and Co., Ltd., £1,000 from the Powell Duffryn Steam Coal Company, and 250 guineas from Mr. David Morgan. £105 will also be received through the Alexandra Rose Day Fund from Sir Edward Nicholl, who recently contributed £10,000 to the hospital.

THE *x*-ray department at the Coventry and Warwickshire Hospital has been presented by the members of the local Women's Liberal Association with a complete combistat, and recently a pleasant function took place, when about thirty of the ladies, along with Mrs. Kirby, the President of the Association, attended at the hospital, when a demonstration was given with the machine by Dr. Webb Fowler and the sister in charge of the department. The working of the *x*-rays was also explained. The deputation also visited the other departments of the hospital. Tea was provided in the nurses' home, when a cheque in payment of the balance of the cost of the apparatus was handed to the chairman of the hospital.

### The Book Market.

LIST OF NEW BOOKS RECEIVED FROM THE FOLLOWING PUBLISHERS:—

Society for the Propagation of the Gospel: "Suffering Relieved." 1s.

Methuen and Co.: "The Care of the Teeth." By Arthur T. Pitts, M.R.C.S., L.R.C.P. 1s. net. "The Drink Problem of To-day." By T. N. Kelynack, M.D. 7s. 6d. net. "The Prevention of the Common Cold." By Oliver K. Williamson, M.A., M.D. 1s. net.

John Wright and Sons, Ltd., Bristol: "The Medical Annual, 1916." 10s. net. "I.K. Therapy in Pulmonary Tuberculosis." By W. Barr, M.D., D.Sc. 3s. 6d. net. "Pye's Surgical Handicraft." Edited and largely rewritten by H. H. Clayton-Greene, B.A., M.B. 15s. net.

Geo. Allen and Unwin, Ltd.: "Newsholme's School Hygiene." New Edition. Rewritten by J. Kerr, M.A., M.D. 4s. 6d. net.

G. Bell and Sons, Ltd.: "Downward Paths: An Inquiry into the Causes which Contribute to the Making of the Prostitute." With a Foreword by A. Maude Royden. 2s. 6d. net.

### THE "SANITAS" COMPANY.

THE annual meeting of this Company, one of the best known and most successful firms which supply and contract for disinfectants and other supplies essential to the efficiency of institutions for the sick, was held on June 28, under the chairmanship of Mr. C. T. Kingzett. The usual dividend of 7½ per cent. was declared, and it was stated that the business of the company has been well maintained, in spite of the unfavourable war conditions. The company has introduced recently a new Sanitas Anti-Vermin Paste, a simple, safe, and preventive remedy for some of the minor horrors of war, which will no doubt prove very popular among soldiers if the belief entertained of its efficacy proves to be well founded, as well as other proprietary articles in the domain of therapeutics.

Rates of Subscription: Twelve months, 6/6; Six months, 4/-; Three months, 2/-, post free to any address in the United Kingdom. Abroad, Twelve months, 8/8; Six months, 5/-; Three months, 2/6, post free. The Hospital "Index" to Volume LIX., October 1915 to March 1916, is now ready, price 1½d. per copy, post free. Address The Manager, "The Hospital," The Hospital Building, 28/29 Southampton Street, Strand, London, W.C.

